# Supplementary material for: Evaluation of transplacental transfer of mRNA vaccine products and functional antibodies during pregnancy and infancy
Source: Nat Commun. 2022 Jul 30;13:4422. doi: 10.1038/s41467-022-32188-1 (PMC9338928; doi:10.1038/s41467-022-32188-1)
Supplement: Supplementary file 1 — Supplementary Information [file 41467_2022_32188_MOESM1_ESM.pdf]

Supplementary Material

Table of Contents

	Page
Table S1	2
Table S2	2
Table S3	3
Figure S1	4
Figure S2	5

Table S1: Maternal Demographics

Study ID	Gestational Age at Delivery	Infant Sex	Vaccine Type	Gestational Age at Dose 1	Maternal Age Category	Days from Dose 1 to Delivery	Days from Dose 2 to Delivery
11012	37.71	Female	Moderna	26.572	35-40	77	58
11018	37.43	Female	Moderna	36	30-34	9	-21
11026	37.57	Female	Moderna	31.858	30-34	36	10
11033	20.43	Male	Pfizer	13.143	25-30	50	27
11036	38.71	Male	Moderna	31	40-44	54	25
11038	39.86	Female	Moderna	31.286	35-30	61	40
11040	39.29	Male	Moderna	33.715	35-40	40	12
11045	38.86	Female	Pfizer	30.572	35-40	58	37
11047	39.29	Male	Moderna	31.572	35-40	54	26
11048	39.14	Male	Pfizer	25.286	30-34	97	75
11058	40.14	Female	Moderna	33.858	30-34	44	23
11059	40.14	Male	Moderna	30.429	35-40	68	47
11062	40.29	Male	Moderna	30.715	35-40	66	39
11063	37.57	Female	Moderna	25	25-30	86	59
11085	41.14	Male	Pfizer	40.286	30-34	6	-15
11091	39.57	Female	Pfizer	28.143	35-40	80	59
11123	39.14	Male	Pfizer	35	35-40	30	9
11126	39.14	Female	Pfizer	36	35-40	45	24
11128	39.71	Male	Pfizer	37	30-34	23	2
11137	40.57	Male	Moderna	37	30-34	36	4

Table S2: Serial dilution of vaccine cDNA

pg Vaccine cDNA	Moderna Ct	Pfizer Ct
10000.00	6.578	6.696
3333.33	8.247	12.266
1111.11	11.403	12.804
370.37	12.799	15.355
123.46	14.327	16.314
41.15	16.217	19.397
13.72	18.430	19.727
4.57	20.503	21.579
1.52	23.315	24.646
0.51	23.337	23.998
0.17	24.259	26.110
0.06	25.510	27.840

*Sensitivity to 1.5 pg/uL

Table S3: Spike protein western blot and vaccine mRNA PCR results

	Spike Protein Detection (Western blot)			Spike mRNA Detection (qRT-PCR)		
Participant	Placenta	Maternal Blood	Cord Blood	Placenta	Maternal Blood	Cord Blood
11012	Negative	Negative	Negative	Negative	Negative	Negative
11018	Negative	Negative	Negative	Negative	Negative	Negative
11026	Negative	Negative	Negative	Negative	Negative	Negative
11033	Negative	Negative	Negative	Negative	Negative	Negative
11036	Negative	Negative	Negative	Negative	Negative	Negative
11038	Negative	Negative	Negative	Negative	Negative	Negative
11040	-	Negative	Negative	-	Negative	Negative
11045	Negative	Negative	Negative	Negative	Negative	Negative
11047	-	Negative	Negative	-	Negative	Negative
11048	Negative	Negative	Negative	Negative	Negative	Negative
11058	Negative	Negative	Negative	Negative	Negative	Negative
11059	-	Negative	Negative	-	Negative	Negative
11062	Negative	Negative	-	Negative	Negative	-
11063	Negative	Negative	Negative	Negative	Negative	Negative
11085	-	-	-	-	-	-
11091	Negative	Negative	Negative	Negative	Negative	Negative
11123	Negative	Negative	Negative	Negative	Negative	Negative
11126	Negative	Negative	Negative	Negative	Negative	Negative
11128	Negative	Negative	Negative	Negative	Negative	Negative
11137	Negative	Negative	-	Negative	Negative	-

- Missing sample

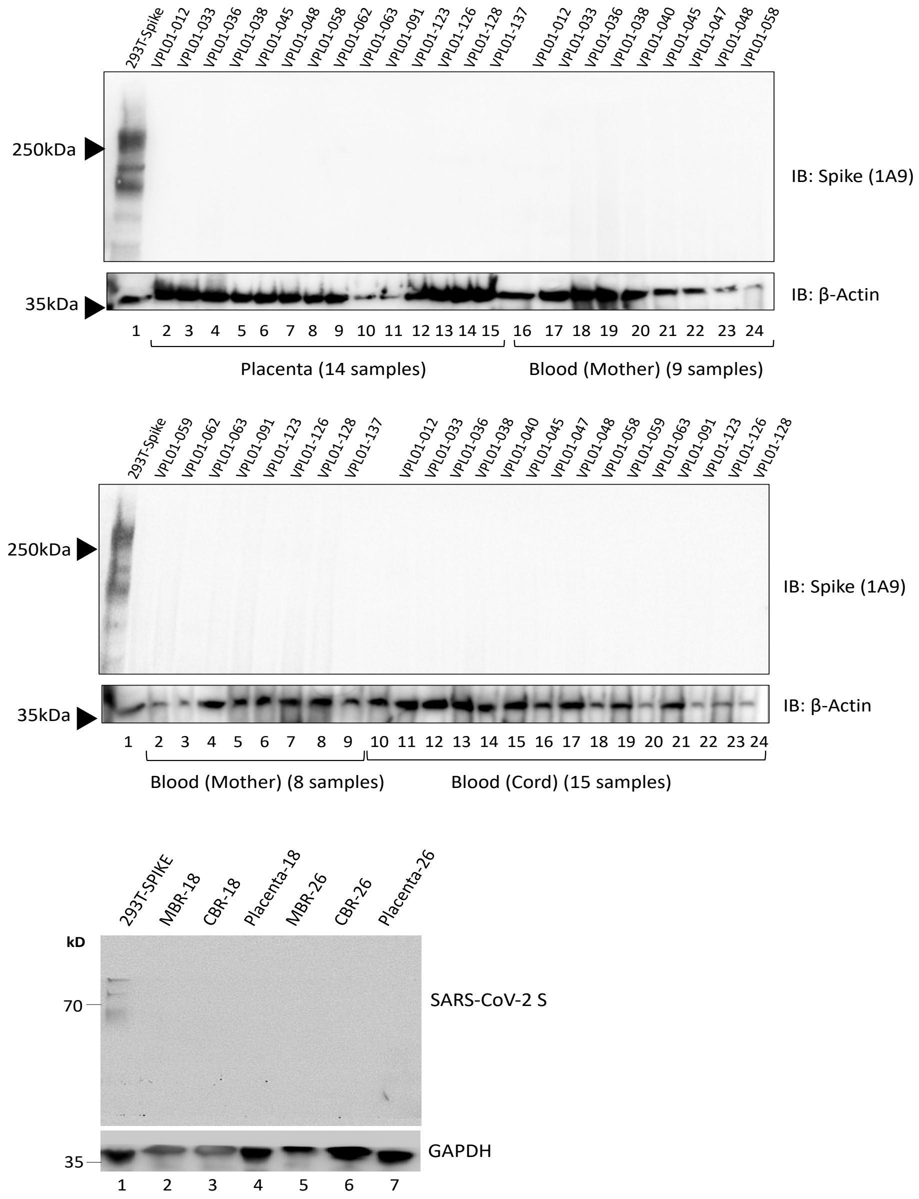

Supplementary Figure 1: Spike protein Western blot

Western blot of maternal blood at delivery, placenta tissue, and cord blood compared to positive control of 293T cells constitutively expressing Spike protein (293T-SPIKE) and labeled with anti-SARS-CoV-2 Spike mouse mAb in single replicate.

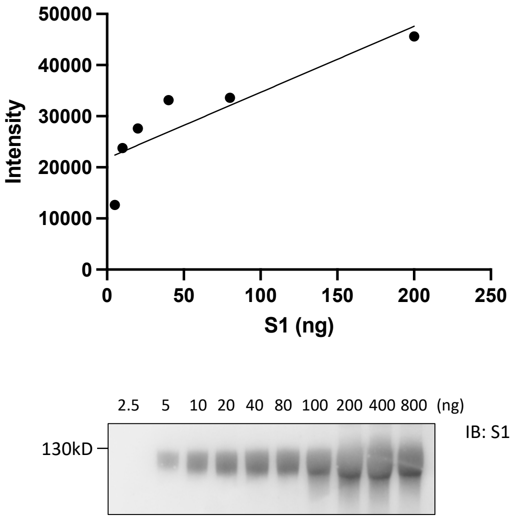

Supplementary Figure 2: Spike protein Western blot standard curve
Western blot was performed using purified S1 protein and the signal intensity of the bands was quantified using a densitometer in single replicate. S1 protein was undetectable at 2.5 ng, and the signal intensity became not linear over 200 ng; the dynamic range of the standard curve was 5ng to 200ng
