# Supplementary material for: Evaluation of transplacental transfer of mRNA vaccine products and functional antibodies during pregnancy and infancy
Source: Nat Commun. 2022 Jul 30;13:4422. doi: 10.1038/s41467-022-32188-1 (PMC9338928; doi:10.1038/s41467-022-32188-1)
Supplement: Supplementary file 3 — Reporting Summary [file 41467_2022_32188_MOESM3_ESM.pdf]

nature portfolio

Corresponding author(s): Mary Prahl, MD
Stephanie Gaw, MD, PhD

Last updated by author(s): Dec 14, 2021

Reporting Summary

Nature Portfolio wishes to improve the reproducibility of the work that we publish. This form provides structure for consistency and transparency in reporting. For further information on Nature Portfolio policies, see our Editorial Policies and the Editorial Policy Checklist.

Statistics

For all statistical analyses, confirm that the following items are present in the figure legend, table legend, main text, or Methods section.

n/a	Confirmed	
☐	☒	The exact sample size (*n*) for each experimental group/condition, given as a discrete number and unit of measurement
☐	☒	A statement on whether measurements were taken from distinct samples or whether the same sample was measured repeatedly
☐	☒	The statistical test(s) used AND whether they are one- or two-sided<br>*Only common tests should be described solely by name; describe more complex techniques in the Methods section.*
☒	☐	A description of all covariates tested
☐	☒	A description of any assumptions or corrections, such as tests of normality and adjustment for multiple comparisons
☐	☒	A full description of the statistical parameters including central tendency (e.g. means) or other basic estimates (e.g. regression coefficient) AND variation (e.g. standard deviation) or associated estimates of uncertainty (e.g. confidence intervals)
☐	☒	For null hypothesis testing, the test statistic (e.g. *F*, *t*, *r*) with confidence intervals, effect sizes, degrees of freedom and *P* value noted<br>*Give P values as exact values whenever suitable.*
☒	☐	For Bayesian analysis, information on the choice of priors and Markov chain Monte Carlo settings
☒	☐	For hierarchical and complex designs, identification of the appropriate level for tests and full reporting of outcomes
☐	☒	Estimates of effect sizes (e.g. Cohen's *d*, Pearson's *r*), indicating how they were calculated

Our web collection on statistics for biologists contains articles on many of the points above.

Software and code

Policy information about availability of computer code

Data collection	QuantaStudio 6 Flex for PCR
Data analysis	PRISM v9.2 (GraphPad), STATA 16 (StataCorp), and R version 3.6.3 and R Studio version 1.1.447, R ggplot2 package

For manuscripts utilizing custom algorithms or software that are central to the research but not yet described in published literature, software must be made available to editors and reviewers. We strongly encourage code deposition in a community repository (e.g. GitHub). See the Nature Portfolio guidelines for submitting code & software for further information.

Data

Policy information about availability of data

All manuscripts must include a data availability statement. This statement should provide the following information, where applicable:

- Accession codes, unique identifiers, or web links for publicly available datasets
- A description of any restrictions on data availability
- For clinical datasets or third party data, please ensure that the statement adheres to our policy

Source data files are provided with this paper (Figures 1-4). The PhIP-Seq/VirScan data generated in this study (Figure 5) have been deposited in the dryad database under accession code: https://doi.org/10.7272/Q6DJ5CWD

Field-specific reporting

Please select the one below that is the best fit for your research. If you are not sure, read the appropriate sections before making your selection.

☒ Life sciences ☐ Behavioural & social sciences ☐ Ecological, evolutionary & environmental sciences

For a reference copy of the document with all sections, see nature.com/documents/nr-reporting-summary-flat.pdf

Life sciences study design

All studies must disclose on these points even when the disclosure is negative.

Sample size	Samples were obtained from a cohort of pregnant individuals enrolled in a study of mRNA vaccination in pregnancy. Sample size was 20 participants based on the first 20 deliveries in the cohort. Sample size was determined by specimen availability and need for rapid information on safety and efficacy of COVID-19 mRNA vaccination due to the lack of enrollment of pregnant individuals in the COVID-19 vaccine clinical trials
Data exclusions	No data was excluded. Subjects were excluded from assays if insufficient specimen was available.
Replication	Each participant sample for mRNA vaccine PCR was run in triplicate. Due to limited sample volume, each participant sample for Spike protein Western blot, anti-SARS-CoV-2 IgM and IgG, SARS-CoV-2 neutralizing assay, and SARS-CoV-2 Spike protein phage array was run as a single replicate. All attempts at replication were successful.
Randomization	All participants received mRNA vaccination independent of this study protocol. Enrollees were not randomized.
Blinding	All participants received mRNA vaccination independent of this study protocol and enrollees were not blinded. Investigators who performed assays were blinded to clinical data prior to performance of experiments.

Reporting for specific materials, systems and methods

We require information from authors about some types of materials, experimental systems and methods used in many studies. Here, indicate whether each material, system or method listed is relevant to your study. If you are not sure if a list item applies to your research, read the appropriate section before selecting a response.

Materials & experimental systems

n/a	Involved in the study	
☐	☒	Antibodies
☐	☒	Eukaryotic cell lines
☒	☐	Palaeontology and archaeology
☒	☐	Animals and other organisms
☐	☒	Human research participants
☒	☐	Clinical data
☒	☐	Dual use research of concern

Methods

n/a	Involved in the study	
☒	☐	ChIP-seq
☒	☐	Flow cytometry
☒	☐	MRI-based neuroimaging

Antibodies

Antibodies used	1) anti-SARS-CoV-2 Spike mouse mAb (1A9, GeneTex), dilution 1:1,000, Cat: GTX632604, clone 1A9, lot#441742 2)anti-GAPDH rabbit polyclonal antibody: catalogue:GTX100118 GeneTex, dilution 1:5,000, lot#. 43747. 3)anti-mouse secondary antibody (GE Healthcare) dilution1:5,000, Cat#NA931, lot#9691073. 4) anti-rabbit secondary antibody (Jackson ImmunoResearch) dilution 1:10,000, Cat# 011-035-144, polyclonal, lot#150324
Validation	1) Datasheet from website (https://www.genetex.com/PDF/Download?catno=GTX632604). From manufacturer's website: This antibody detects both SARS-CoV spike and SARS-CoV-2 spike proteins (S2 subunit). Our internal testing indicates no cross-reactivity with MERS-CoV spike protein. This antibody is able to detect multiple SARS-CoV-2 VOCs, including Omicron variant. Additional publications with use of antibody: PMID: 35104835, PMID: 34982967. 2)Datasheet from website (https://www.genetex.com/PDF/Download?catno=GTX100118). Additional publications with use of antibody: PMID: 35296653, PMID: 35317026 3) https://www.cytivalifesciences.com/en/us/shop/protein-analysis/blotting-and-detection/blotting-standards-and-reagents/amersham-ecl-hrp-conjugated-antibodies-p-06260. 4) https://www.jacksonimmuno.com/catalog/products/111-035-144

Eukaryotic cell lines

Policy information about cell lines

Cell line source(s)	Positive control of 293T cells constitutively expressing wild-type SARS-CoV-2 Spike protein. Gladstone Institute/Greene lab produced
Authentication	None of the cells lines were authenticated.
Mycoplasma contamination	Cell lines tested negative for mycoplasma contamination
Commonly misidentified lines (See ICLAC register)	None

Human research participants

Policy information about studies involving human research participants

Population characteristics	We enrolled 20 female pregnant women and their infants who were vaccinated with either BNT-162b2 or mRNA-1273 mRNA vaccines independent of this study protocol. Participant age, infant sex, and gestational age at vaccination were recorded and are documented in Table S1.
Recruitment	Participants who received mRNA vaccination between December 2020 and April 2021 were recruited by physician-referral and self-referral. Due to the CDC initial recommendations on prioritization of the initial vaccine roll out recipients, many of the early participants were front-line workers and healthcare workers in later stages of pregnancy which may contribute to self-selection bias and limited evaluation of participants in early phases of pregnancy. No participants received financial compensation for study enrollment.
Ethics oversight	The University of California San Francisco (UCSF) institutional review board approved the study (20-32077).

Note that full information on the approval of the study protocol must also be provided in the manuscript.
