# Supplementary figures and images for: Evaluation of transplacental transfer of mRNA vaccine products and functional antibodies during pregnancy and infancy
Source: Nat Commun. 2022 Jul 30;13:4422. doi: 10.1038/s41467-022-32188-1 (PMC9338928; doi:10.1038/s41467-022-32188-1)

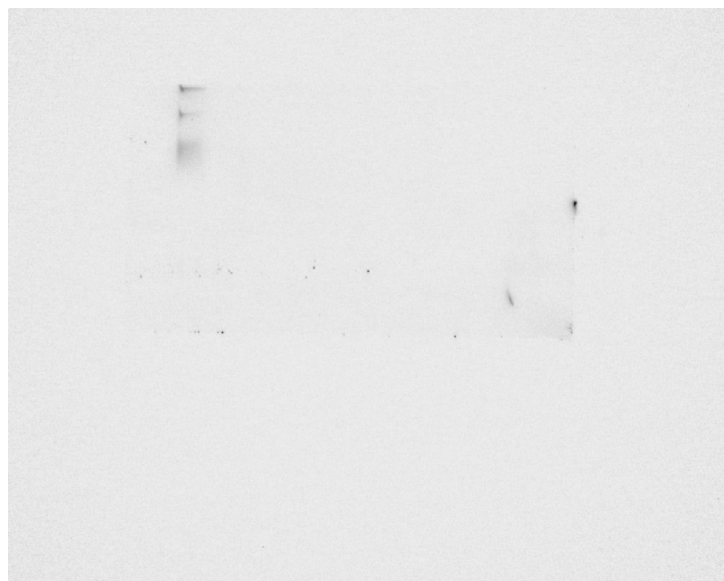

Supplement: Supplementary file 4 — Source Dataset [file 41467_2022_32188_MOESM4_ESM.zip › Nature Comm Source data/Source data.SARS-CoV-2 S blot.pdf]

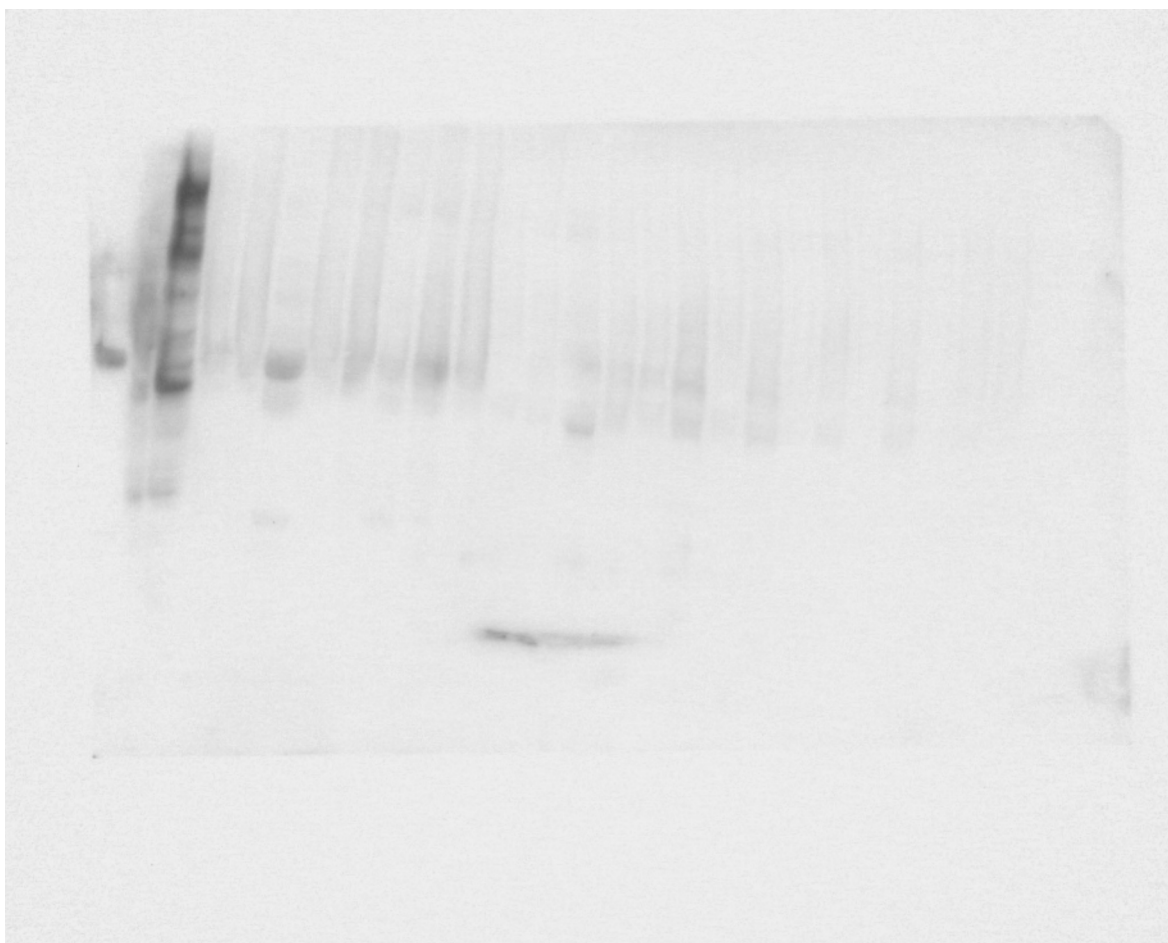

Supplement: Supplementary file 4 — Source Dataset [file 41467_2022_32188_MOESM4_ESM.zip › Nature Comm Source data/Source data.Spike 1A9.2 blot.pdf]

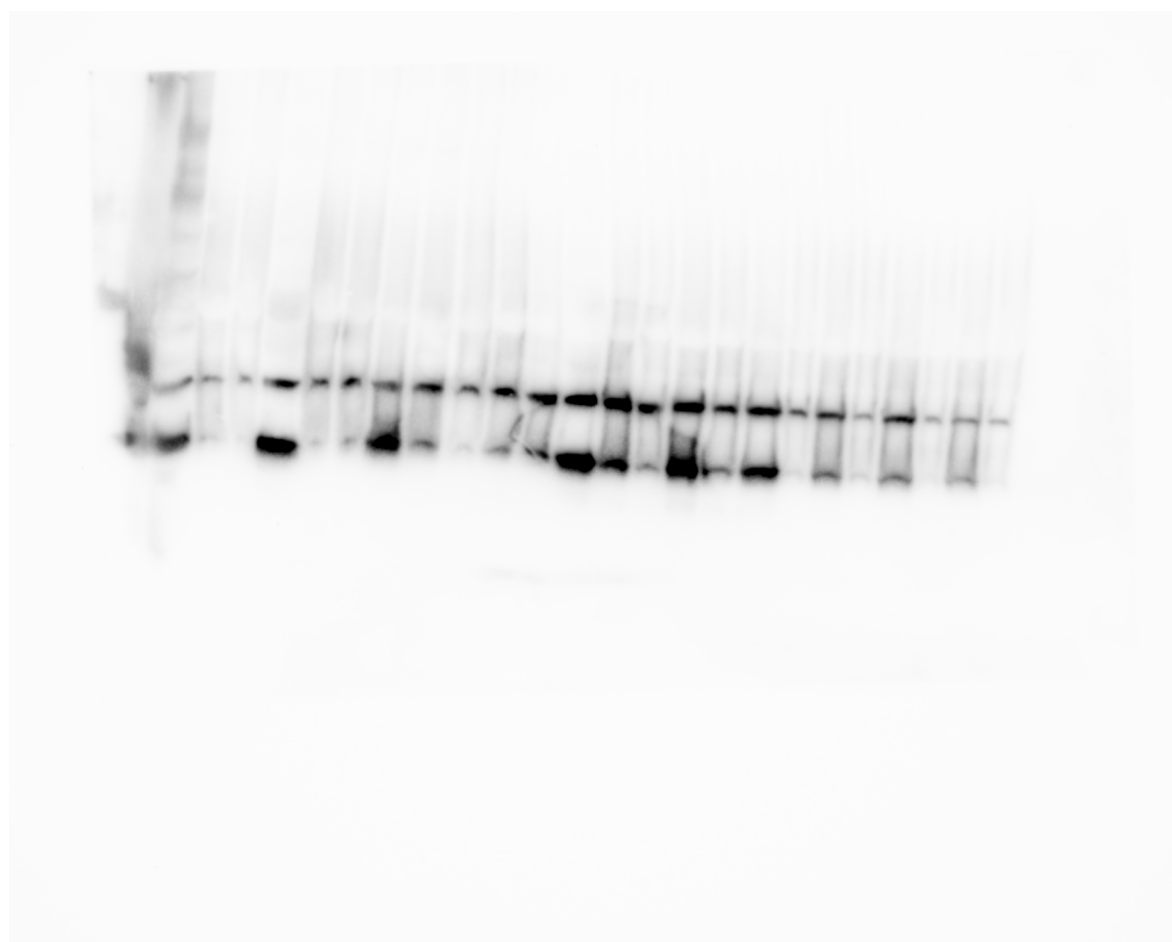

Supplement: Supplementary file 4 — Source Dataset [file 41467_2022_32188_MOESM4_ESM.zip › Nature Comm Source data/Source data.beta actin.2 blot.pdf]

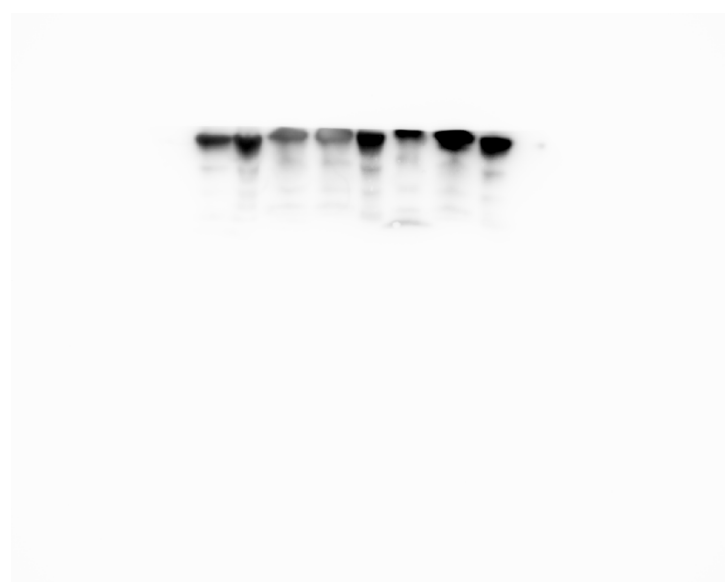

Supplement: Supplementary file 4 — Source Dataset [file 41467_2022_32188_MOESM4_ESM.zip › Nature Comm Source data/Source data.GAPDH blot.pdf]

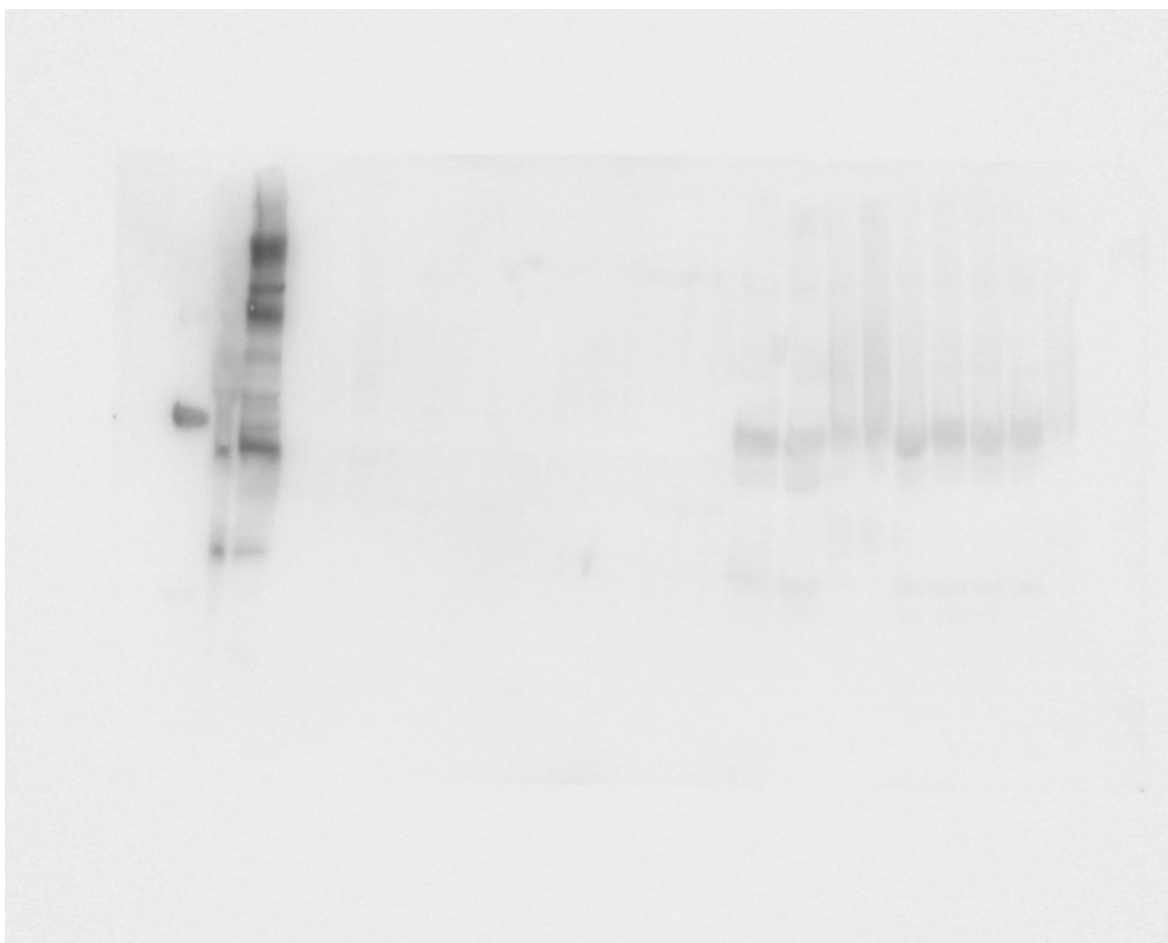

Supplement: Supplementary file 4 — Source Dataset [file 41467_2022_32188_MOESM4_ESM.zip › Nature Comm Source data/Source data.Spike 1A9.1 blot.pdf]

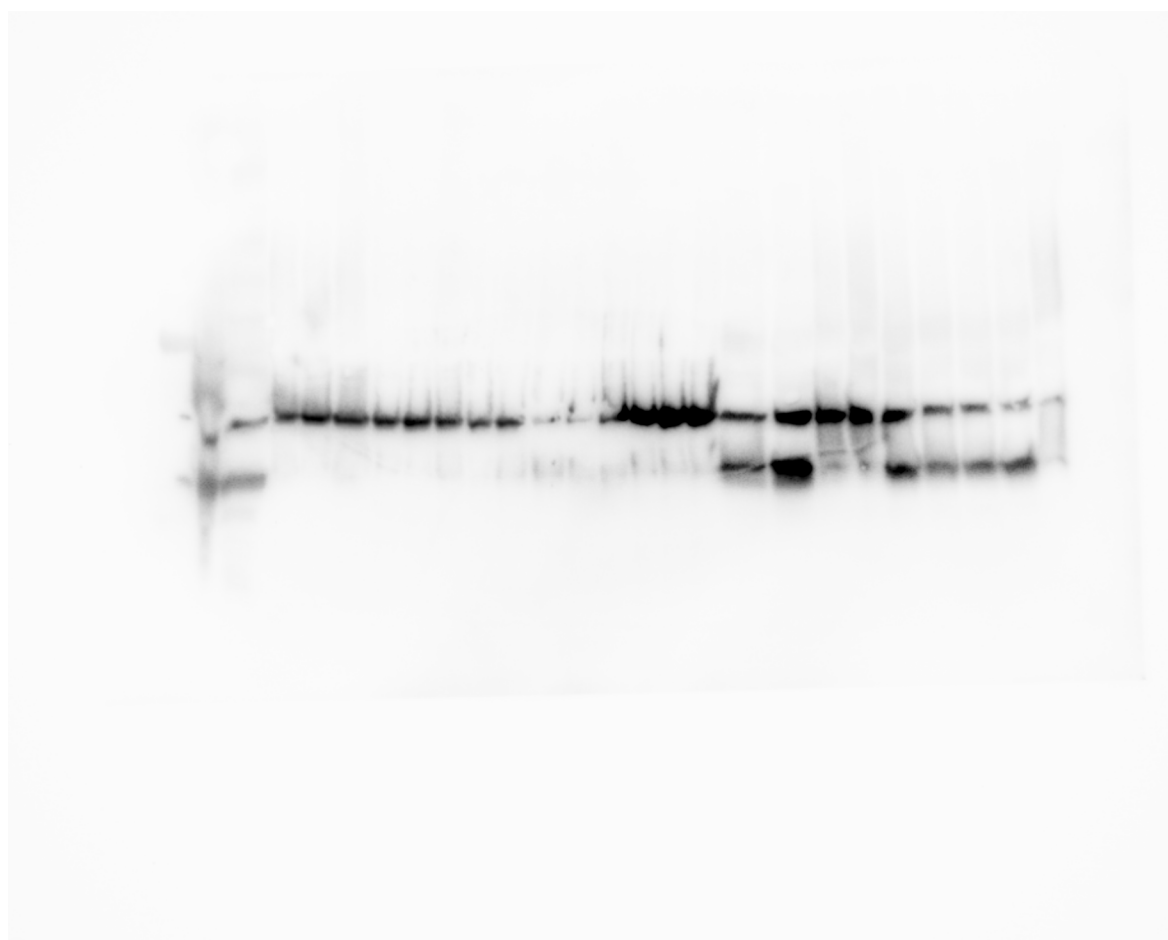

Supplement: Supplementary file 4 — Source Dataset [file 41467_2022_32188_MOESM4_ESM.zip › Nature Comm Source data/Source data.beta actin.1 blot.pdf]
